# How humans adapt to hot climates learned from the recent research on tropical indigenes

**DOI:** 10.1186/s40101-022-00302-3

**Published:** 2022-07-14

**Authors:** Yutaka Tochihara, Hitoshi Wakabayashi, Joo-Young Lee, Titis Wijayanto, Nobuko Hashiguchi, Mohamed Saat

**Affiliations:** 1 Department of Human Science, Faculty of Design, Kyushu University, 4-9-1 Shiobaru Minami-ku, Fukuoka, 815-8540 Japan; 2 Laboratory of Environmental Ergonomics, Faculty of Engineering, Hokkaido University, N13 W8, Kita-ku, Sapporo, Hokkaido 060-8628 Japan; 3 Department of Textiles, Merchandising and Fashion Design, College of Human Ecology, Seoul National University, 1 Gwanak-ro, Gwanak-gu, Seoul, 08826 Korea; 4 Laboratory of Ergonomics, Department of Mechanical and Industrial Engineering, Faculty of Engineering, Universitas Gadjah Mada, Jl Grafika 2 Kampus UGM, Yogyakarta, 55281 Indonesia; 5 Department of Health Science, Faculty of Medicine, Kyushu University, 3-1-1 Maidashi, Higashi-ku, Fukuoka, 812-8582 Japan; 6 School of Medical Sciences, Health Campus, Universiti Sains Malaysia, 16150 Kubang Kerian, Kelantan Malaysia

**Keywords:** Human heat adaptation, Tropical and temperate indigenes, Thermal sensation, Cutaneous thermal sensitivity, Decay of acclimatization, Cognitive function, Body temperature and fluid regulation

## Abstract

This review mainly aimed to introduce the findings of research projects comparing the responses of tropical and temperate indigenes to heat. From a questionnaire survey on thermal sensation and comfort of Indonesians and Japanese, we found that the thermal descriptor “cool” in tropical indigenes connotes a thermally comfortable feeling, suggesting that linguistic heat acclimatization exists on a cognitive level. Ten male students born and raised in Malaysia were invited to Fukuoka, Japan, and compared their responses with 10 Japanese male students with matched physical fitness and morphological characteristics. Cutaneous thermal sensitivity: The sensitivities were measured at 28 °C. The forehead warm sensitivity was significantly blunted in Malaysians. The less sensitivity to the warmth of tropical indigenes is advantageous in respect to withstanding heat stress with less discomfort and a greater ability to work in hot climates. Passive heat stress: Thermoregulatory responses, especially sweating, were investigated, during the lower leg hot bathing (42 °C for 60 min). The rectal temperature at rest was higher in Malaysians and increased smaller during immersion. There was no significant difference in the total amount of sweating between the two groups, while the local sweating on the forehead and thighs was lesser in Malaysians, suggesting distribution of sweating was different from Japanese. Exercise: Malaysian showed a significantly smaller increase in their rectal temperature during 55% maximal exercise for 60 min in heat (32 °C 70% relative humidity), even with a similar sweating and skin blood flow response in Japanese. The better heat tolerance in Malaysians could be explained by the greater convective heat transfer from the body core to the skin due to the greater core-to-skin temperature gradient. In addition, when they were hydrated, Malaysian participants showed better body fluid regulation with smaller reduction in plasma volume at the end of the exercise compared to the non-hydrated condition, whereas Japanese showed no difference between hydration conditions. We further investigated the de-acclimatization of heat adaptation by longitudinal observation on the heat tolerance of international students who had moved from tropical areas to Fukuoka for several years.

## Background

Human beings thought to have been born in the tropics of Africa about 7 million years ago gradually expanded their living space to lands with cold climates through biological and cultural adaptation. Finally, they reached all over the earth about 10,000 years ago. Currently, human beings have overcome the harsh natural environment with the power of science and technology, gradually expanding their range of survival and even creating more comfortable artificial environments. The artificial environment created by highly developed science and technology has provided a place where people can live comfortably by freely manipulating various living environment factors, such as thermal, light, air quality, and sound environments. Moreover, we are expanding our activity to the oceans and space, places that 100 years ago, human beings could only dream of inhabiting.

However, a significant problem is whether human beings can physiologically adapt to such a new artificial environment. In other words, the artificial environment in which modern humans spend most of their day is not healthy. Instead, it goes against the biological characteristics of human beings, and there is a risk of human health being impaired [1]. The artificial environment created by technology often focuses on the pursuit of convenience and an improved economy. It is not designed based on the physiological and psychological characteristics of human beings. In the case of the thermal environment, for example, overcooling by the air-cooling system in the summer causes “cooling disease.” It cannot be denied that the artificial thermal environment has various adverse effects. It is feared that the expansion of the artificial thermal environment in the last 100 years may deteriorate the ability of human beings to adapt to the natural thermal environment acquired over hundreds of thousands of years. These thermal adaptation problems were one of the main research topics for physiological anthropologists [2–4].

Many studies on the adaptation to heat and cold have been conducted by Japanese physiologists [5, 6]. Internationally, the International Biological Program (IBP) was an advanced global research project on human-environmental adaptability. IBP was planned in Europe in 1964 [7] and adopted by the Science Council of Japan. Physiologists, hygienists, geneticists, and anthropologists attended IBP under “The biological basis of productivity and human welfare.” One of the purposes of this program was to “examine the adaptability of human beings to various gradually changing environmental conditions.” The research was based on “population growth,” “food shortage,” and “urbanization.” It was predicted that as urbanization progressed, humankind’s physical strength and adaptability would change due to changes in the living environment [8]. Currently (50 years after the IBP), the artificial environment and global warming have become more serious problems. The use of air-conditioning system has rapidly progressed. Most building are air-conditioned; many homes are being installed with an air-conditioning system. Thus, people spend all year under comfortable temperature conditions. On the other hand, recently, there have been concerns about global warming. It is necessary to raise or lower the temperature setting of cooling and heating as much as possible to save energy. For example, there is recommended temperature setting at 28 °C for cooling (Cool Biz) and 20 °C for heating (Warm Biz) in Japan. However, the accurate physiologically target values and applicability remain unclear.

There are two ways that human beings can deal with global warming today: (1) using an artificial environment (cooling devise) and (2) expecting human beings’ inherent adaptability to heat. When choosing the latter, it is essential to know the physiological characteristics of tropical indigenes with excellent heat tolerance during heat exposure. There were many studies on regional differences in heat tolerance on a global scale [9–16]. However, these studies on heat tolerance experiments for tropical and temperate indigenes were often conducted at different locations. And the physical fitness and physiques of the two groups were sometimes different. It is known that humans with higher physical fitness [17] and a larger surface area-to-mass ratio [18] have greater resistance to high temperatures. Therefore, there was concern that the physiological differences during heat exposure between the two groups might be due to the differences in measurement procedures and the differences in the physical fitness and physiques of the subjects. In addition, the duration of stay at the experimental site varied considerably from experiment to experiment. The length of stay of subjects in temperate or hot areas might affect the physiological response during heat exposure. The most distinctive feature of this study to solve these concerns was that we conducted experiments on regional differences in heat tolerance on a global scale by inviting tropical indigenes to Fukuoka. Thus, the measurement locations, measuring devices, and measuring techniques used were the same. We also matched the morphological characteristics and physical fitness of the participants from tropical and temperate regions in the series of studies.

Furthermore, we could elucidate the mechanism of de-acclimation of heat adaptation by longitudinally observing the heat tolerance of international students who had moved from tropical areas to Fukuoka for several years. Moreover, even if heat adaptation is successful, intellectual productivity decline may significantly impact economic activity. Therefore, the limit of adaptability to heat without deteriorating intellectual productivity was determined by measuring the cerebral blood flow during heat performance of the subject with a tissue oxygen monitor using near-infrared spectroscopy. We further investigated whether semantic differences in different languages express thermal sensation and comfort between tropical and temperate indigenes.

In order to investigate the abovementioned issues, we conducted a joint international project. The key person of this project was Dr. Mohamed Saat from the Universiti Sains Malaysia. He was initially researching the heat adaptation of sweating under Professor Mitsuo Kosaka of the Institute of Tropical Medicine, Nagasaki University [19], and subsequently received a Ph.D. on “heat adaptation of tropic-dwelling people” study [16, 20–22] at Kyushu University using the JSPS RONPAKU program (dissertation Ph.D.). The project aimed to compare and examine regional differences in the thermal adaptability of tropical indigenes and Japanese. Ten Malaysian students from Kota Bharu were invited to Kyushu University for 2 weeks to undergo various thermal experiments. They were compared with 10 Japanese students from Kyushu University living in Fukuoka. The latter matched their physical work capacity and physique. The average annual temperature and relative humidity (RH) of Kota Bharu and Fukuoka were 27 °C, 80%, and 17 °C, 70%, respectively. The time difference between the two cities is 1 h.

The purpose of this review was to introduce the project which was intended to compare and examine regional differences in thermal adaptability of tropical indigenes and Japanese at the same laboratory using the same experimental equipment and technique. We also examined the decay of heat tolerance of tropical indigenes. Moreover, we investigated cognitive function in heat and whether there were semantic differences in different languages expressing thermal sensation and comfort between tropical and temperate indigenes. This project will be expected to provide a clue as to how to prevent the decline in the ability of temperate people to adapt to heat because it could be physiological proposals for long-term adaptation to combat global warming and the spread of artificial thermal environments.

Most of the experimental studies presented in this review article were conducted at the Research Center for Human Environmental Adaptation of Kyushu University in Fukuoka, Japan This research center was initially established by Professor Masahiko Sato in 1971 and was completely reconstructed in 2001. It is situated in a two-story building containing nine climatic chambers that control air pressure, air temperature, air humidity, air velocity, illumination, and water pressure (Table 1 in Appendix). This center is a leading international research center in the field of human environmental adaptation.

### Thermal sensation

When a person is exposed to heat, thermal receptors are stimulated in the body, and heat sensation is perceived. From thermal stimuli imposed on the body, physiological effector mechanisms operate, and eventually, thermal sensation can be cognized through words and concepts in the mind. Typically, scales for measuring thermal sensation are categorical scales, for example, cold, cool, slightly cool, neutral, slightly warm, warm, and hot [23, 24]. In this section, it is important to distinguish thermal sense, itself, from thermal sensation perceived through the brain. Thermal sense is universal for humans irrespective of nationality or ethnicity, whereas perceived thermal sensation expressed by language is not universal [24]. We found that the thermal descriptor “warm” of the 7-point-verbal ASHRAE scale and ISO 10551 [25] did not correspond with Japanese and Korean’s conscious thermal feelings in terms of the thermal descriptor “warm” (“Atatakai あたたかい” and “Ttatthada 따뜻하다”).

Japanese and Korean languages are typically classified as the Altaic language family and share the same sentence structure (subject + object + verb) and similar vocabulary. Japanese and Korean words that are respectively translated as “warm” in English are “atatakai” and “ttatthada,” which do not exactly correspond to “warm” of the ASHARE scale. Japanese and Korean words for “warm” express a thermally positive feeling. For Koreans and Japanese people, to explicitly express “feeling warm” is to implicitly connote a thermally comfortable state [26]. Pitts [27] stated that, in translation, phrases may come to have different meanings than their original, especially when the translation is into a very different language. Humphreys [28] compared the ASHRAE thermal sensation scale in the following languages of “English, French, Greek, Portuguese, and Swedish” and concluded that these words have different meanings depending on which language they are being used in. In this respect, Lee and Tochihara [29] surveyed on Korean verbal descriptors expressing thermal sensation and found that “warm” in Korean projects thermal comfort (in 80.4% of the 988 respondents).

It is likely that the diversity of thermal sensation descriptors is proportional to the diversity of thermal environments in a culture. In the process of constructing thermal sensation scales for Indonesian and Malaysian languages, we hypothesized that the appropriate thermal sensation adjectives would reflect the thermal adaptive traits of the people living in these particular tropical climates. We explored whether or not there is evidence of heat acclimatization in their words used to express thermal sensation in Indonesian [30]. A total of 601 Indonesians participated in a survey, and we found that, for Indonesians, the closest thermal descriptor of a feeling of thermal comfort was “cool” (75% of the 601 respondents). In cases where “cool” was imagined in the mind, the descriptor was cognized as a thermally comfortable feeling by 97% of the Indonesians. We, thus, concluded that the thermal descriptor “cool” in Indonesian connotes a thermally comfortable feeling. This suggests that linguistic heat acclimatization exists on a cognitive level for Indonesians and is preserved in the words of thermal descriptors.

Studies with similar interests have been reported. In 2016, Damiati and colleagues conducted a survey in Malaysia, Indonesia, Singapore, and Japan during hot and humid season and found that thermal descriptors “cool” and “warm” in Japanese have positive meanings [31]. Next year, Khatun et al. [32] will published an international survey with 1141 university students from Bangladesh and Japan, using the ASHRAE 11-point scale. They reported that the Bangladesh students responded “neutral” and “cold” as “thermal comfort,” which reflects the heat acclimatization effect. The exact semantic meaning of these words does not correspond perfectly to the ASHRAE definitions. In 2018, an interesting study was published by Al-Khatri and Gadi [33] which proposed that eastern Arab students translated “cool” into Arabic with words that literally means “moderate,” “mild,” or “neither cool nor warm.” This is a neutral understanding of cool. The following studies support our hypothesis on linguistic acclimatization to heat.

Since the Indonesian study, there have been suggested worldwide questionnaire including residents in colder climate zones as well. Recently, a large-scale international survey translated into 21 languages from 26 countries was developed based on 8225 questionnaire respondents [34]. The goal of the study was to investigate possible differences in the interpretation of thermal sensation scales due to climate and language. Respondents were classified into subgroups according to relationships between thermal sensations and comfort. For example, subgroup 1’s comfort was associated with verbal anchors on the warm side, while thermal comfort of subgroup 5 was associated with feelings of cool or cold. However, for subgroup 5, verbal anchors on the warm side were placed as being uncomfortable or extremely uncomfortable. Subgroup 1 consisted of mostly those from “Cf” (temperate climate) in the Köppen-Geiger climate classification with the least number of those from Af (tropical rain forest) or Aw (tropical Savannah). Subgroups 4 and 5 were most frequent in Af and Aw, and subgroup 5 was characterized by hot and humid climates (e.g., Malaysia). Schweiker and colleagues [34] found that people residing in hot climates assigned comfort to thermal sensations on the cold side, whereas people who resides in temperate and colder climates tended to assign comfort to thermal sensations ranging from “neutral” to “slightly warm.”

Our original concept of linguistic heat acclimatization could be a valuable tool for physiological anthropologists exploring the relationships between climate and language. Based on Lee and Tochihara [29], Tochihara et al. [30], and Schweiker et al. [34], one may suppose that Koreans today use the thermal descriptor “warm” only for feeling thermally comfortable due to the fact that ancient Koreans lived in a colder climate. However, further comprehensive surveys incorporating more types of indigenes living in cold or subarctic climates (e.g., Mongolians) as well as tropical climates are required to prove this proposition.

### Cutaneous thermal sensitivity

Cutaneous thermal receptors transmit information about the temperature of the skin [35, 36] and then mediate the autonomic temperature regulation as well as generate thermal comfort through the integration of thermal sensations [37, 38]. Perceived thermal sensation drives our thermoregulatory behavior, such as controlling air-conditioning systems, changing clothes and postures, drinking water, and seeking comfortable shelter. These thermoregulatory behaviors extend our homeostatic range in hostile environments beyond the range provided by internal physiological reactions.

In the previous section, we introduced our studies dealing with a hypothesis on linguistic heat acclimatization in terms of thermal sensation descriptors. In this section, we introduce some studies on heat acclimatization and cutaneous thermal sensitivity as a psychophysiological adaptive trait. In fact, the effect of heat acclimatization on thermal perceptive responses is a relatively neglected area of research when compared to research on physiological adaptation. Regarding cutaneous thermal perception, it has been reported that there is a noticeable reduction in thermal discomfort after repeated exposure to heat or cold [39–41], which can be called “habituation.” Elucidating ethnic differences in thermal perception is of particular interest in individual variations. Inter-individual variations in physiological responses have been explained by acclimatization, as well as physical fitness, hydration state, drug and alcohol use, gender, anthropometric data, age, etc. [42–44]. Differences in thermal perception modified by acclimatization are also of particular interest for this research.

**Fig. 1:**
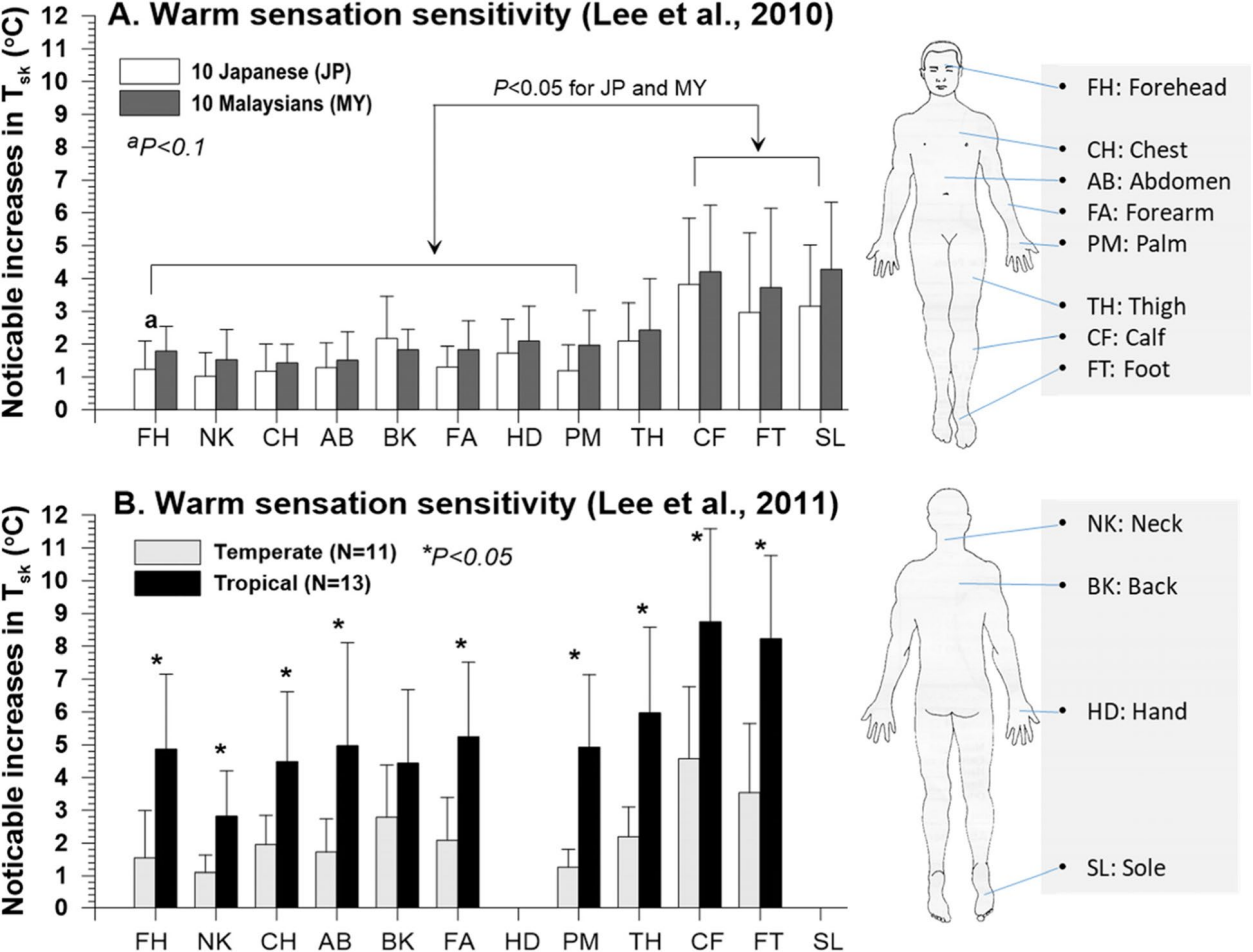
Increases in skin temperature (*T*_sk_) to detect initial warm sensation on the 10 to 12 body regions of tropical and temperate indigenes. Values indicate mean and SD. **A** and **B** have been redrawn from Lee et al. [45] and Lee et al. [46], respectively

With this background, we investigated differences in cutaneous thermal sensitivity and the inter-threshold sensory zones between tropical (10 Malaysians) and temperate indigenes (10 Japanese) (Fig. 1A) [45]. We found that Malaysians tended to perceive warmth at a higher skin temperature and more slowly than Japanese, and they also had wider ranges for the inter-threshold sensory zones on the skin than Japanese. Malaysians’ wider inter-threshold sensory zones were mainly due to their wider warm thresholds rather than cool thresholds. These results were verified even for tropical indigenes who had resided in temperate climates for ∼5 years (Fig. 1B) [46].

Adaptation is a characteristic of most sensory systems and describes the decrease in neural responsiveness of primary afferents to a stimulus due to sustained contact with that stimulus [47]. One could ask whether tropical indigenes’ lower sensitivity to warmth perception is due to a “deforming modification” or due to an “improved modification.” The reduction of cutaneous thermal sensitivity due to aging [48–52] is an example of a deforming modification, whereas the gradual diminishing of sensory responses and discomfort after repetitive exposures [39, 53] is an improved modification. The lower sensitivity to warmth of tropical indigenes is advantageous for their ability to withstanding heat stress with less discomfort and greater ability to work and function in hot climates. One criterion to draw a boundary between the deforming and improved modification would be a “reversibility.” The improved modification is reversible. Phenotype adaptation means a change in a reversible manner [54].

Comparative physiologists consider acclimation to be a reversible response [55]. The rate of decay in heat acclimation is also affected by the number of heat exposures per week [56]. Compared with studies on the decay of heat acclimation (short term and artificial heat), the decay in heat acclimatization (long term and natural heat) has received relatively little attention due to methodological limitations. Because knowledge of de-acclimatization of cutaneous thermal sensitivity in humans is lacking, we conducted a second study in order to examine whether or not the de-acclimatization of the cutaneous thermal sensations of tropical indigenes residing in temperate climates exists [46]. In the study, we recruited 13 tropical indigenes (Caucasians, Asians, and Africans; lean to overweight subjects) who were born and raised in tropics but had resided in Japan for 5–61 months and found that the tropical indigenes were, on average, 3.3 °C and 3.5 °C less sensitive to warm and hot stimuli, respectively, than temperate indigenes. The inter-threshold sensory zones between cutaneous warm and cool sensations were wider for the tropical than for the temperate indigenes. As a follow-up study, we explored seasonal variations in the cutaneous thermal perception of tropical indigenes who reside in Japan [57]. The cutaneous thermal sensitivity of the tropical indigenes (7 male students) were retained after the residence of at least 42 months in Japan without any significant variation between four seasons, while Japanese students (11 males) had greater sensitivity to cold in summer than in winter. These results indicate that the essential nature of the cutaneous warm sensitivity of tropical indigenes is retained after their prolonged residence in a temperate climate for at least about 4–5 years.

Most physiologists assume that heat acclimatization is at least a partially phenotypic adaptation [18]. Reversible changes in sweat onset time and sweat rate have been documented for tropical indigenes residing in moderate climates for extended periods of time [58]. Changes in sweating activity or skin blood flow from heat adaptation was detected in temperate indigenes who had lived in the tropics for less than 2 years [59], a mean period of 18 months [39], less than 3 years [10], or more than 2 years [60]. These results suggest that the decay or increase in sweating function or skin vascular activity from heat acclimatization is manifested within a few years.

Cats that were subjected to temperatures of 5 °C for 2 months had no indication of a functional changes in facial cutaneous receptors [61], whereas cats that had been living in the cold for 4 years displayed functional changes in their cutaneous receptors [62]. Considering the previous findings during the decay of heat acclimation (short term adaptation), heart rate changes occur first followed by a reduction in core temperature and then adjustments in the sweat rate [63–65]. These observations suggest that the desensitization of thermal sensitivity is a heat exposure time-dependent phenomenon. Also, it has been proposed that changes in cutaneous thermal sensitivity are induced by alterations in the structure and function of cutaneous thermal receptors [66], such as decreases in the number or size of warm/cold receptors in the skin, changes in the neurotransmission speed of nerves, and a reduced maximum firing activity of single cold-responsive units [67, 68]. Furthermore, about 50 genes display altered expression levels during exposure to heat stress [69]. If the heat acclimatization of cutaneous thermal sensitivity is involved in integrated alterations across several body levels, this would contribute to more robust retention of heat adaptive traits.

In this section, we introduced novel and interesting findings from a series of studies on the cutaneous thermal sensitivity of tropical and temperate indigenes, but there are certain limitations to these studies, especially their methodologies. Many factors underlie the measurement of thermal thresholds as covariates: methods (method of limit vs. method of level), type of heating modes (contact vs. radiant vs. immersion), adaptation temperature (cold vs. neutral vs. warm), rate of temperature increase (0.1, 0.5, 1, or 2 °C/s), stimulated surface area (from just a few millimeters to more than 10 cm^2^), age (the young vs. the elderly), ancestry (European vs. African American), gender (male vs. female), total body fat (the lean vs. the obese), skin type (hairy vs. glabrous skin), etc. Higher cutaneous warm thresholds are associated with a faster rate of temperature increase, smaller stimulated area, and the elderly [70, 71]. All values should be interpreted with these types of methodological considerations.

### Thermoregulatory responses of tropical and temperate indigenes to heat

#### Resting baseline in a thermoneutral environment

**Fig. 2:**
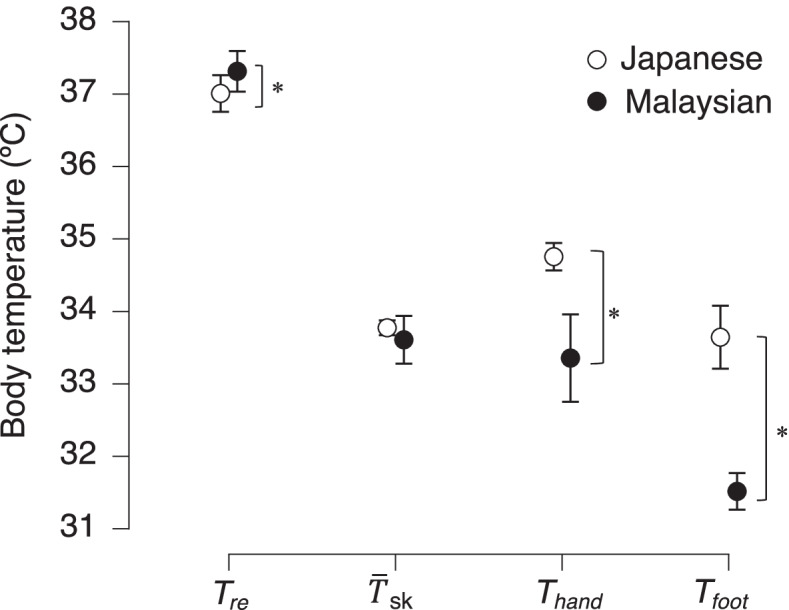
Rectal temperature, mean skin temperature, hand temperature, and foot temperature during resting in thermoneutral condition (air temperature of 28 °C and air humidity of 50% RH). Values  indicate mean and SE. *Asterisk* shows significant difference between group at *p* < 0.05. Data obtained from Lee et al. [76]

Earlier research found that people of tropical climates such as those from Singapore [72], India [73], and Vietnam [74, 75] had significantly higher average core temperatures (*T*_core_) at rest. Saat et al. (2005) also found that tropical indigenes from Malaysia had a higher resting rectal temperature (*T*_re_) than temperate indigenes from Japan. Several heat-load studies have revealed these findings as heat adaptation features of the indigenous from tropical climates. In a series of heat adaptation studies, Lee et al. [76] observed that Malaysians had higher *T*_re_ than Japanese after 70-min resting in a lying position in a climatic chamber with 28 °C and 50% RH (Fig. 2). This finding is consistent throughout the series of our experiment. Preconditioned *T*_re_ was higher in Malaysians before they had passive [77] or active heat exposure [78]. These findings reinforced higher *T*_core_ in tropical indigenes as reported in the earlier studies [20, 72–74, 79]. Interestingly, the higher resting *T*_core_ observed in tropical indigenes, with long-term heat acclimatization, is opposed to the well-known findings in the studies on short-term heat acclimation, which reported lower resting *T*_core_ after heat acclimation [80–82]. Lee et al. [76] considered that Malaysians had a higher resting *T*_re_ to reduce heat dissipation through vascular changes rather than increased heat production or less evaporative cooling. While resting *T*_re_ showed ethnic difference, no difference was observed in resting mean skin temperature ($\overline{T}$_sk_) between Malaysian and Japanese. Interestingly, in addition to having a higher resting *T*_re_ with no different $\overline{T}$_sk_, Lee et al. [76] also reported that the Malaysians had cooler hand and foot temperature (*T*_hand_ and *T*_foot_, respectively) than Japanese (Fig. 2). Higher resting *T*_re_ and lower *T*_hand_ and *T*_foot_ may be a preconditioned state to reduce thermal and cardiovascular strains during passive heat exposure.

Although ethnic differences were found in body temperature, insensible body mass loss did not significantly differ between the two groups. The same insensible body mass loss level indicates the similar level of evaporative heat loss in a thermoneutral condition. Furthermore, Lee et al. [76] discovered a positive correlation between insensible body mass loss and rectal temperature change. The more significant the insensible body mass loss, the greater the fall in *T*_re_ during resting for both Malaysians and Japanese. This correlation indicates that insensible perspiration significantly influences rectal temperature stability.

#### Passive heat stress

**Fig. 3:**
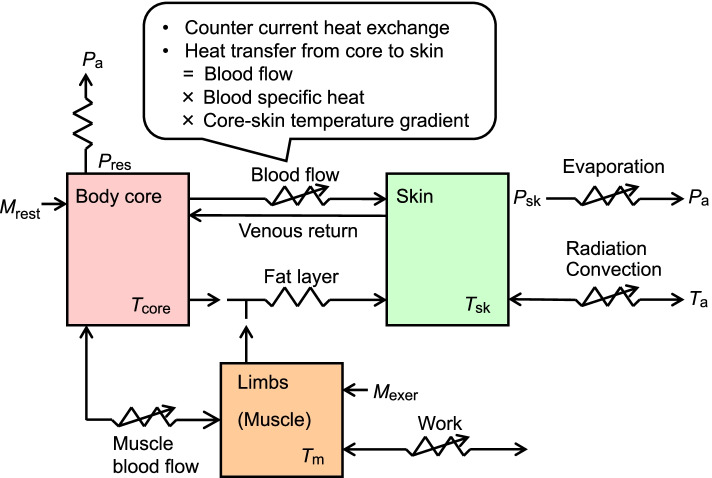
Scheme of heat transfer pathway from human core body to the environment. *T*_core_, *T*_sk_, *T*_m_, and *T*_air_ represent temperature of body core, skin, muscle, and ambient air. *P*_res_, *P*_sk_, and *P*_a_ are water vapor pressure at respiratory tract, skin surface, and ambient air. *M*_rest_ and *M*_exer_ are metabolic heat production at rest and during exercise. The scheme was modified from Nadel et al. [84]

Similar to the findings in the resting in thermoneutral experiment, Wijayanto et al. [77] observed higher preconditioned *T*_re_ and lower skin temperature at the extremities (*T*_hand_ and *T*_foot_) in Malaysians. Taylor et al. [83] suggested that a higher pre-exposure *T*_core_ might enhance convective heat dissipation as the core-to-skin temperature gradient increase. The scheme of the convective heat transfer pathway from the human core body to the environment is summarized in Fig. 3. The smaller rise in *T*_core_ indicates higher internal stability or homeostasis in dealing with heat stress. These differences were then diminished after the Malaysians and Japanese immersed their lower legs to the knees into a 42 °C warm water for 60 min. No significant differences in *T*_re_ and *T*_hand_ between these two groups were found. Consequently, the increase of *T*_re_ (*ΔT*_re_) was smaller in Malaysians than in Japanese. We extended this study by comparing Japanese students and tropical students from Southeast Asia, who had lived in Japan for 1 year [85] and 2 years [86] in average. These studies observed the same findings, a higher preconditioned *T*_re_ with a smaller *ΔT*_*re*_ during leg immersion in Southeast Asians. Their findings corroborate earlier studies reporting a smaller core temperature rise during leg immersion in tropical individuals [14, 79].

Despite the lower rise in *T*_re_, Malaysians reported higher elevations in *T*_hand_ during leg immersion [76, 77]. There are at least two probable explanations of the suppression of *ΔT*_re_ in Malaysians. First, *ΔT*_re_ might be slowed when 2 °C colder blood from the hands mix with the warmer core blood. Thus, we observed a smaller *ΔT*_re_ in Malaysians after leg immersion. Second, Wilder’s law of initial value might contribute to the suppression of rectal temperature during leg immersion. Lee et al. [76] tested the law of initial value on *T*_re_ and *T*_hand_ results. They found that higher resting *T*_re_ in Malaysians was associated with a smaller *ΔT*_re_. Meanwhile, lower resting *T*_hand_ was associated with a more significant increase in *T*_hand_. These findings reflect the homeostatic nature of organisms with physiological restrictions as the ceiling value and imply that tropical indigenes have a more thermally stable core and thermally plastic extremities than temperate indigenes.

**Fig. 4:**
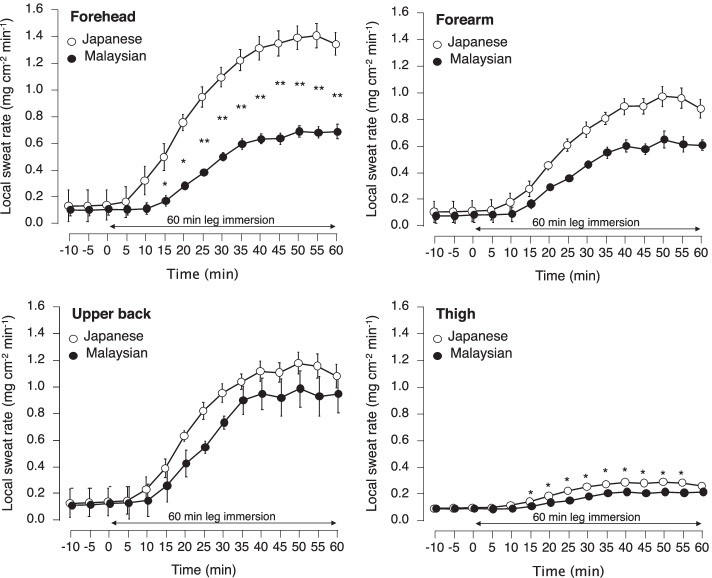
Local sweat rate on the forehead, upper back, forearm, and thigh during 60-min leg immersion. Values are mean and SE. *Asterisk* indicates a significant difference at *p* < 0.05. Redrawn from Wijayanto et al. [77]

Another finding of leg immersion study comparing Malaysians and Japanese was that Malaysians had lower local sweat rates on the forehead and thigh (Fig. 4). Both Malaysians and Japanese had no significant differences in total and local sweat rates on the upper back and forearm [77]. Earlier studies reported that tropical indigenes sweat less than nontropical indigenes [12, 14, 16, 87]. Wijayanto et al. [77] confirmed this notion regarding local sweat responses, not the whole-body sweating response. The distribution of local sweat rate during leg immersion might be different between Malaysians and Japanese. The local sweat rate in Malaysians could be similar or higher than in Japanese. The Malaysian subjects sweat more uniformly over the body or on the limbs than Japanese subjects. When the sweat distribution is uniform, the wet body surface increases, offering an advantage in evaporative sweat loss in a hot and humid environment. We considered that the uniform distribution of sweating in those tropical natives might be most likely due to adaptive adaptation to a hot and humid environment.

#### Exercise: active heat stress

In addition to the resting and passive heating condition, we compared thermoregulatory response in temperate and tropical indigenes during exercise in hot and humid environments (32 °C, 70% RH) [78, 88]. The heat stress during exercise protocol was higher than that in passive heating protocol, which enabled us to clarify the advanced body temperature regulation in the tropical indigenes with intense heat strain. The participants from temperate and tropical countries were matched with similar morphological characteristics and peak oxygen uptake (VO_2peak_) as well. Thus, the absolute and relative exercise intensity at 55% of their maximal intensity was both comparable for assessing the difference in their thermoregulatory responses at the same heat strain.

The increase in *T*_re_ during 60-min exercise was smaller in tropical group compared to temperate group [78, 88], which suggested that tropical indigenes have better heat tolerance with some advantage in their body temperature regulation. Regarding sudomotor function, no ethnical difference was observed in their local sweat rate or total body weight loss. This result was opposed to the earlier study reporting less local sweat rate in tropical natives during exercise in thermoneutral environment [89]. However, in line with our finding, many previous studies reported no difference in total sweat loss [13, 16, 73, 90]. The intense heat stress during exercise in hot environment would maximize the sudomotor response in tropical indigenes as well as temperate counterparts. Regarding vasomotor function, the skin blood flow relative to the baseline and mean skin temperature at the end of the exercise was lower in tropical group. Lower blood perfusion in tropical indigenes was also described in several studies during exercise in heat [13, 90, 91]. It would indicate less heat dissipation from the skin in tropical natives, which seems to be inconsistent with the result of smaller increase in their core body temperature. To explain the potential mechanism of the better heat tolerance in the tropical indigenes, the scheme of heat transfer pathway from human core body to the environment is summarized in Fig. 3 (modified from [84]). Passive heat conduction and convective heat transfer in blood circulation between the body core, skin, and limbs (working muscle) are depicted. We added to the original scheme an arrow for venous return from the skin to body core and description of counter current heat exchange and convective heat transfer from core to the skin (Fig. 3). The advanced body temperature regulation observed in the tropical participants would be due to the lower hand skin temperature and higher core body temperature at rest, as explained in the studies of resting baseline and passive heat stress [76, 77]. The lower baseline hand-skin temperature in tropical participants might play a role as cold-heat reservoir for suppressing the rise of core body temperature through the counter current heat exchange between cooler venous return from extremities and warmer arterial blood flow. During active heat stress, tropical indigenes increased their hand-skin temperature more than the temperate group [78, 88]. It suggested that tropical indigenes might have a specific economic distribution of blood to their hands with greater surface area to mass ratio and large number of arteriovenous anastomoses [92]. Additionally, there is a process of heat transfer from core body parts to the skin through the blood circulation (Fig. 3), which is a product of core-skin temperature gradient, blood flow, and the volume-specific heat of blood [83]. The tropical group in our study showed greater core-skin temperature gradient throughout the exercise protocol, partly due to the higher resting core body temperature, which was systematically observed in studies on other tropical populations [73–75, 79, 85, 90]. During exercise in heat, blood perfusion to the skin and exercising muscle are simultaneously elevated and can become competitive between these organs [93]. Thus, the convective core-to-skin heat transfer due to the core-skin temperature gradient has an advantage for maintaining central blood volume and muscle blood flow, especially during long duration exercise in heat.

**Fig. 5:**
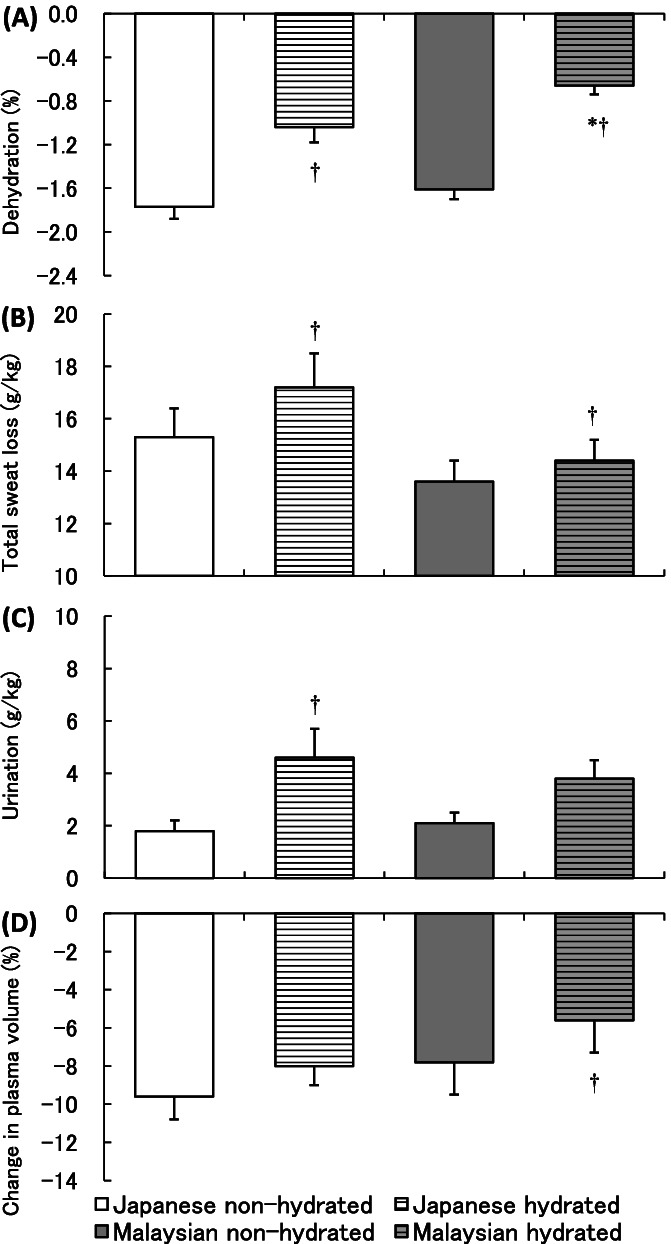
**A** Percentage of dehydration, **B** total sweat loss, **C** urine loss per unit body weight, and **D** percent change in plasma volume. Values are mean and SE. *Asterisk* indicates significant difference at *p* < 0.05 between groups. Dagger indicates a significant difference at *p* < 0.05 between conditions. Data obtained from Wakabayashi et al. [94]

For further understanding long-term heat acclimatization in the blood volume control, we conducted additional study to compare the effect of hydration on body fluid and temperature regulation between tropical and temperate indigenes exercising in heat [94]. Both groups performed exercise for 60 min at 55% VO_2peak_ followed by 30-min recovery in hot and humid environment (32 °C, 70% RH) with water intake relative to their body weight (12 mL/kg in total) or without hydration. Tropical group with hydration showed a significantly lower *T*_re_ at the end of 60-min exercise compared to non-hydrated condition, whereas no hydration effect was observed in *T*_re_ of temperate group. This result indicated that tropical indigenes better utilized the limited amount of water intake for a heat dissipation response. Regarding the body fluid regulation, the percentage in dehydration (body weight loss including the ingested water) during the protocol was significantly less in Malaysians than in Japanese in the hydrated condition, whereas no group difference was observed for the non-hydrated condition (Fig. 5A). Total sweat loss was significantly more in the hydrated condition for both groups and tended to be less in hydrated Malaysians than in hydrated Japanese (*p* = 0.08, Fig. 5B). In addition, the amount of urination per body weight was increased with hydration in Japanese but not in Malaysians (Fig. 5C). According to these results, tropical indigenes could suppress dehydration with minimizing wasteful overproduction of sweat and urine. The advantage of body fluid conservation in Malaysians resulted in a significantly smaller reduction of plasma volume after 60-min exercise in the hydrated condition than in non-hydrated condition, whereas no difference was observed in Japanese with or without hydration (Fig. 5D). The less body fluid loss and smaller reduction in plasma volume in tropical indigenes with hydration indicated an advantage in body fluid conservation. This might enable tropical natives to reserve more circulating blood for heat dissipation and thereby maintain lower core body temperature when they are hydrated. In fact, a significant interaction of groups and hydration conditions in cutaneous vascular conductance was observed during exercise, which indicated that hydration could enhance skin blood flow in tropical indigenes but not in the temperate group.

### Decay of heat acclimatization

Unlike heat acclimatization, heat acclimation improves heat tolerance by lowering resting and exercising heart rates, resting core temperature, and increasing perspiration rate [80–82, 95–97]. Lind [98] suggested that heat acclimation gains heat tolerance for 2 weeks, but it will quickly fade away if not maintained by recurrent heat exposure. Decay of heat acclimation is believed to proceed rapidly [63, 65], with 1 day of adaptation lost for every 2 days spent without exposure to heat [99], or decays every 2 days without work and will vanish 6–21 days after acclimatization [65, 100]. Additionally, it is believed that the adaptations that occur initially, such as an increase in plasma volume and a decrease in exercise heart rate, will dissipate the fastest [63–65, 101]. The percentage decay of heat acclimation for sweat and heart rates is more significant than core temperature [63, 65, 100]. While studies on the decay of heat acclimation are well-documented, study on heat acclimatization decay is relatively rare.

The tropical indigenes have been regarded to be adapted to heat. However, the heat adaptation features of this population may decay after living in a relatively colder environment. According to an earlier study by Saat et al. [19], tropical indigenes from Malaysia who had lived in the temperate area for more than 27 months, such as Japan, had earlier sweating onset time than those who had lived less than 15 months. Lee et al. [58] also discovered that Malaysians who lived in Japan for 2 to 72 months showed a gradual loss of heat acclimatization in their sweating onset time and sweat volume indicated by the change in sweat response to acetylcholine iontophoresis.

**Fig. 6:**
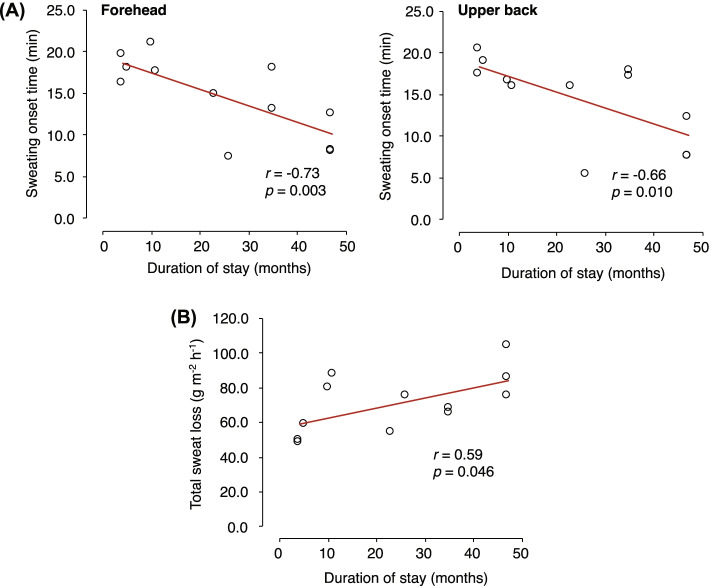
**A** Relationships between duration of stay in Japan and sweating onset time on the forehead and upper back. **B** Relationship between duration of stay in Japan and total sweat rate during 60-min leg immersion. Data obtained from Wijayanto et al. [86]

A study by Wijayanto et al. [86] evaluated the thermoregulatory responses to passive heat exposure in tropical indigenes from Indonesia, Vietnam, Thailand, Philippines, and Malaysia who had stayed in the temperate country of Japan. Their study observed decay of heat acclimatization in the tropical natives as indicated by the changes in sweating responses to heat exposure in tropical indigenes signifying time-dependent characteristics. Total sweat loss increased, and sweating onset time shortened as the duration of stay in Japan increased (Fig. 6). These findings corroborate earlier studies reporting changes in sweating reaction after prolonged residence in the temperate area [19, 58]. Other physiological responses, such as higher resting rectal temperature and smaller increased in rectal temperature, remained unchanged as the period of stay in Japan increased. Wijayanto et al. [86] suggested that the evidence of heat acclimatization in tropical natives did not decay even though they stayed in a colder climate. Their study suggested that heat tolerance acquired from heat acclimatization may be retained. Although it partially decays, it does not entirely diminish during a residence for less than 4 years in a temperate area.

### Cognitive function

While most heat acclimation studies concentrated on physiological responses to heat, relatively few studies were concerned with cognitive functioning in heat. Radakovic et al. [102] reported that heat acclimation prevented cognitive performance degradation during heat exposure. Their findings indicated that acclimatized individuals with better physiological function to tolerate heat stress should be more resistant to performance losses than non-acclimatized individuals. As mentioned earlier, tropical natives from southeast Asians had a better heat tolerance and capacity to maintain homeostasis during heat exposure than temperate natives due to heat acclimatization [76–78]. Wijayanto et al. [85] investigated whether heat acclimatization also results in their ability to maintain cognitive performance in the heat. They compared the cognitive functioning under heat stress in tropical indigenes from Southeast Asia (Indonesia, Malaysia, Philippines, Vietnam, and Thailand) and temperate indigenes from Japan. Both groups reported no performance decrement on a simple task (short-term memory task) during heat exposure. This finding agreed with the previous study reporting the non-detrimental effect of heat exposure on short-term memory [103]. The subjects appeared to try maintaining performance during passive heat exposure. Razmjou and Kjellberg [104] suggested a compensatory system of effort allocation to govern and maintain performance under thermal stress. During task performance in heat, oxyhemoglobin changes (OxyHb) in the prefrontal cortex increased [85, 103], indicating brain metabolic activation to support cognitive demand during a cognitive task [105]. Higher OxyHb levels may indicate brain resource recruitment to balance the increasing cognitive demand to maintain performance level in stressful conditions [85, 103, 106].

**Fig. 7:**
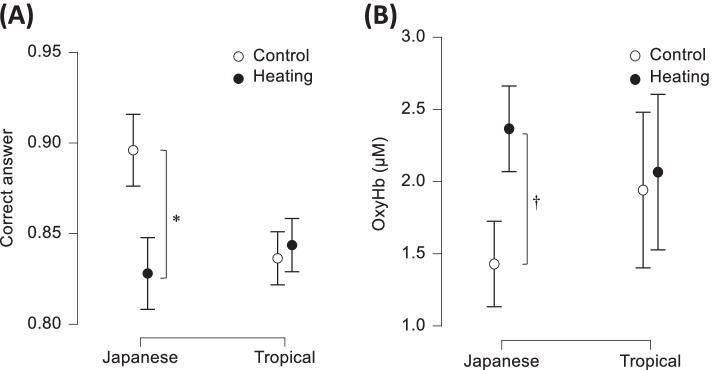
**A** Cognitive task performance of two-column digit addition test. **B** Concentration changes of oxyhemoglobin (OxyHb) during control and passive heating condition. Values are mean and SE. *Asterisk* indicates significant difference at *p* < 0.05. *Dagger* indicates a tendency toward *p* < 0.1. Data obtained from Wijayanto et al. [85]

Contrary to short-term memory, Wijayanto et al. [85] reported performance decrement in a mental arithmetic task (two-column digit addition task) in the Japanese group, not in the tropical group (Fig. 7A). The Japanese subjects in their study reported that heat exposure was hotter and more uncomfortable than tropical subjects. Gaoua et al. [107] suggested that thermal discomfort may impair cognitive function and alter a complex task performance in the heat. Thermal discomfort and fatigue, which are higher during exposure to a hot environment, combined with task complexity, may lead to performance deterioration in Japanese group during heat exposure. Furthermore, the Japanese group also had a higher prefrontal OxyHb level before and during task performance in heat, indicating they expended more neural resources in their brain than the tropical native group (Fig. 7B). Despite having a higher OxyHb level, individuals in the Japanese group were unable to maintain their mental arithmetic task performance. Prior to executing the tasks during heat exposure, we noticed that the Japanese group had already a higher OxyHb than the tropical group. Higher OxyHb levels prior to the task with heat exposure may indicate that the brain resources in Japanese prior to task performance were already overloaded, and the arousal level was too high [103]. A higher arousal level in Japanese likely impaired task performance. An increase in arousal level may improve cognitive function [108, 109], whereas an over increase in arousal level may degrade performance [110]. The results of this investigation showed that heat-acclimatized individuals (tropical Asians) would be more resistant to performance losses during heat stress than non-acclimatized individuals (Japanese). Based on these findings, we considered that the tropical individuals could physiologically endure any heat exposure and retain their cognitive performance with less cognitive effort than the Japanese due to long-term heat adaptations in the tropical climate.

## Conclusions and future work

In this review article, we summarized the previous and recent findings on the topics of long-term heat acclimatization in tropical indigenes, including thermal sensation, cutaneous thermal sensitivity, body temperature, and fluid regulations and cognitive functions. These knowledge of the advanced heat tolerance features in tropical indigenes will contribute to future projects including physiological anthropology, health and life science, industrial hygiene, standardization of thermal environment, and global environmental policy.

Variation in human beings has been one of the hottest topics in the research fields of physiological anthropology. Among the factors of human variation, the adaptability to the thermal environment is the best essential element to consider our living and working environment connected with the history of human great journey. The series of our studies on tropical indigenes focused mainly on their physiological and psychological features under controlling of morphological characteristics. In future, we have to further challenge clarifying human adaptation as a complex system considering interaction among factors including physiological, anthropometric, and genetic variations. Considering the variation in human adaptation to thermal environment, some modification should be conducted for improving the current regulation or standards for thermal environment. For instance, international standards for predicting thermal sensation and comfort in moderate thermal environment [111], assessment of heat stress using the WBGT (wet-bulb globe temperature) index [112], and assessment of the influence of the thermal environment using subjective scales [25] would need some correction factors and/or specific note for target people with long-term heat acclimatization. Especially as global warming progresses, risk management of heat stroke during working in hot environment is becoming more and more important for occupational health. The findings in our latest research on heat acclimatization would be suggestive for preventing heat illness and would be applied to environmental ergonomics, including the designing of personal protective equipment [113, 114] with consideration of thermal insulation and evaporative resistance [115]. Further research progress would be expected to deepen our understanding of human adaptability to hot environment based on the current knowledge summarized in this review article.

## Data Availability

All data generated or analyzed during this study are included in this published article.

## Appendix

Table 1

**Table 1:** Performance capacities of nine climatic chambers at the Research Center for Human Environmental Adaptation (URL: http://www.id.design.kyushu-u.ac.jp/ninkou/english/homot.html)

	Air temperature	Relative humidity	Illumination	Remarks
**No. 1** Hyper and hypobaric chamber with lavatory and shower room	−10~50 ± 0.5 °C	30~80 ± 5%	0~2,000 lx	Air pressure 253~4052 ± 5 hPA O_2_ 10~40 ± 0.1%
**No. 2** Illumination chamber	0~50 ± 0.2 °C	30~90 ± 2%	0~20,000 lx Color temperature (3,000~10,000 k)	
**No. 3** Combined factor chamber	0~50 ± 0.2 °C Wall and floor temperature (10~45 ± 1 °C)	10~90 ± 2%	0~17,000 lx	Air velocity 0~1.5 m/s for each of three layers
**No. 4** Thermal chamber	−40~50 ± 0.2 °C	20~90 ± 3%	0~1,500 lx	
**No. 5** Living environment chamber with bathroom, lavatory, and kitchen	0~40 ± 0.2 °C	30~80 ± 2%	0~2,000 lx Color temperature of artificial window (3,000~10,000 K)	
**No. 6** Water immersion chamber	−10~50 ± 0.5 °C	30~80 ± 3%	1,200 lx	Depth 0~5 m Temp. 5~45 °C
**No. 7** Thermal radiation chamber with bathroom and lavatory	−10~50 ± 0.5 °C	30~80 ± 3%	1,500 lx	Thermal radiation 0~1.000 W/m^2^
**No. 8** Noise reduction and radio shield chamber	0~50 ± 0.2 °C	30~80 ± 3%	0~2,000 lx	Electric shield
**No. 9** Noise reduction and radio shield chamber	0~50 ± 0.2 °C	30~80 ± 3%	0~2,000 lx	Electric shield

## Abbreviations

ASHRAE: American Society of Heating, Refrigerating and Air-Conditioning Engineers
IBP: International Biological Program
ISO: International Organization for Standardization
JSPS: Japan Society for the Promotion of Science
OxyHb: Oxyhemoglobin
RH: Relative humidity
*T*_core_: Core temperature
*T*_foot_: Foot skin temperature
*T*_hand_: Hand skin temperature
*T*_re_: Rectal temperature
$\overline{T}$_sk_: Mean skin temperature
VO_2peak_: Peak oxygen uptake
WBGT: Wet-bulb globe temperature
